# Potential Role of Contact Pathway Factors in Catheter-Related Thrombosis: Emerging Evidence and Therapeutic Strategies

**DOI:** 10.3390/biom16091256

**Published:** 2026-08-29

**Authors:** Mingyan Jin, Chunliang Liu, Song Lyu, Aoxue Li, Kesheng Dai, Jun Wan

**Affiliations:** Cyrus Tang Medical Institute, Jiangsu Institute of Hematology, NHC Key Laboratory of Thrombosis and Hemostasis, Collaborative Innovation Center of Hematology, National Clinical Research Center for Hematological Diseases, Soochow Medical College, Soochow University, Suzhou 215123, China; 2330802014@stu.suda.edu.cn (M.J.); 13646207261@163.com (C.L.); 20244253018@stu.suda.edu.cn (S.L.); 2330506027@stu.suda.edu.cn (A.L.)

**Keywords:** catheter, thrombosis, contact pathway, coagulation

## Abstract

The catheter is among the most commonly used blood-contacting medical devices, but its use can induce surface-mediated coagulation activation, leading to catheter-related thrombosis (CRT). The occurrence of CRT causes venous thromboembolism and catheter malfunction, but current antithrombotic strategies have unsatisfactory efficacy and safety profiles. Here, we review recent advances in the understanding of the pathology of CRT, particularly the roles of the contact pathway factors, and promising novel therapeutic options. Recent studies using genetically modified animals, factor-deficient plasmas, specific inhibitors and purified systems demonstrated an important contribution of contact pathway factors XII and XI to catheter-related blood clotting. Accordingly, contact pathway inhibition has efficacy comparable to that of heparins in mitigating catheter-related coagulation or intraluminal occlusion in various in vitro and animal models, while having lower bleeding risk. Early human studies suggest potential thromboprotective effects of FXI inhibition in catheter placement and hemodialysis settings. However, inhibition of factors XII or XI may impair the defense against infection or disturb normal cardiac function, respectively. Larger human trials are needed to further confirm the efficacy and safety of these contact pathway inhibitors, and to explore whether low-dose combinations of contact pathway inhibitors with heparins are more effective for CRT protection.

## 1. Introduction

**Figure 1:**
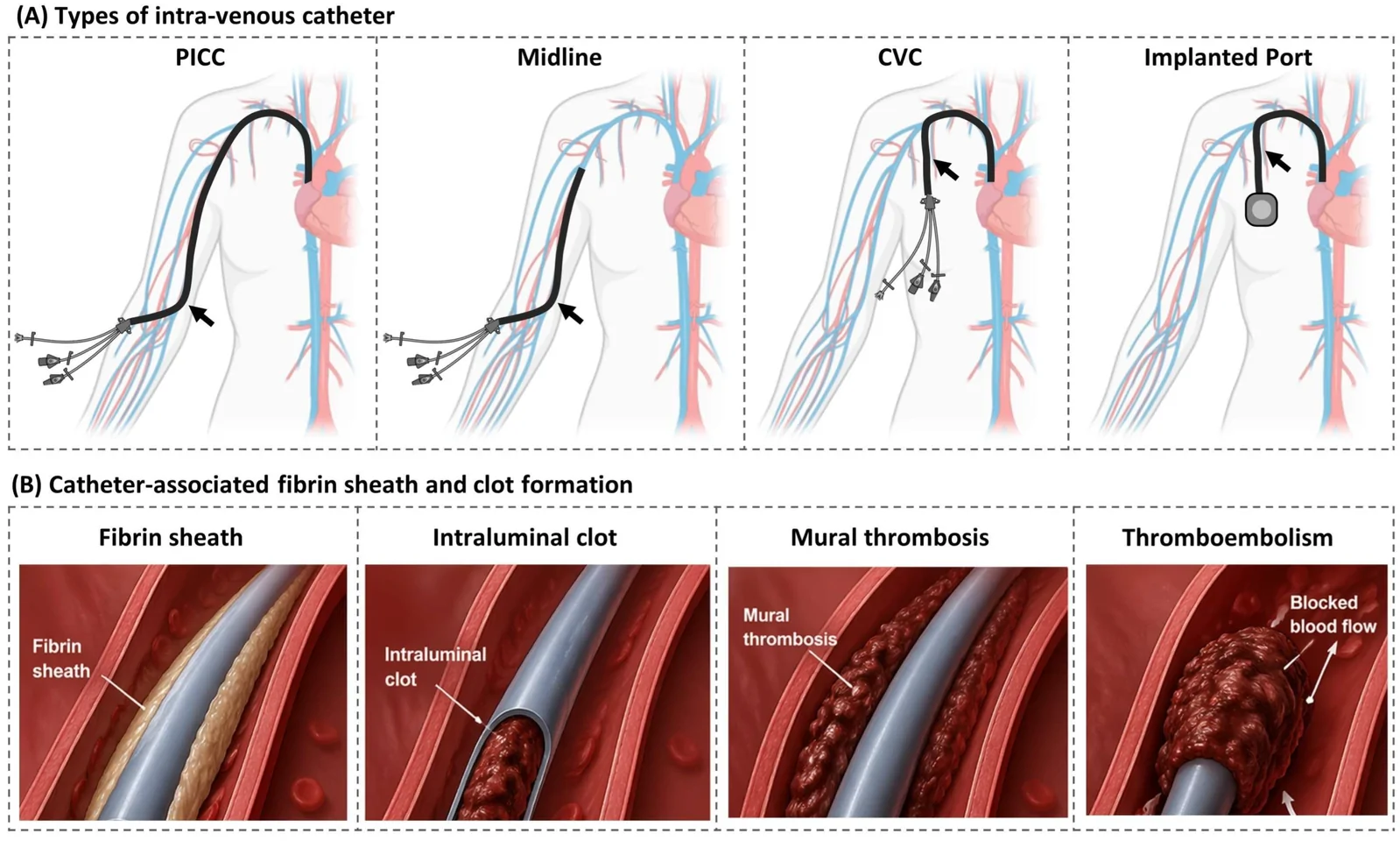
Illustration of common types of intravenous catheters and catheter-associated blood clotting, intraluminal catheter occlusion and thromboembolism. (**A**) Common types of intravenous catheter: (1) peripherally inserted central catheter (PICC), which is inserted through a vein in the upper arm and ends in larger veins near the heart; (2) midline catheter, which is inserted through a vein in the upper arm, with tip located at or near the level of the axilla and distal to the shoulder; (3) central venous catheter (CVC), which is placed through the internal jugular vein or subclavian vein and ends in larger veins near the heart; (4) sometimes the CVC is connected to an implanted port that is placed under the skin in the chest, arm, or abdomen. The arrows indicate insertion sites. (**B**) Catheter placement can lead to activation of the coagulation system and fibrin formation, leading to fibrin sheath deposition on the catheter, intraluminal catheter occlusion, mural thrombosis, or thromboembolism. Schematic diagram created using Nano-banana, with modifications.

The clinical use of blood-contacting medical devices is severely challenged by side effects, including thrombosis, i.e., medical device-associated thrombosis (MDAT) [1]. Such devices include cardiovascular implants such as catheters, extracorporeal membrane oxygenation (ECMO) circuits, and hemodialysis circuits, as well as mechanical heart valves and stents. Catheters are among the most commonly used blood-contacting surfaces, widely employed for infusion therapy, hemodialysis, blood transfusion, and central venous pressure monitoring [2,3]. Catheter-related thrombosis (CRT) is a common complication of catheter use in clinical practice [3], defined as thrombus formation within the vein housing the catheter. The typical pathological feature is a mural thrombus that adheres to the venous wall and extends along the catheter [4], as depicted in Figure 1. CRT poses serious risks to patient safety, including deep vein thrombosis (DVT) and pulmonary embolism (PE). Additionally, CRT can cause catheter dysfunction and reduce catheter durability [5].

Studies report that the incidence of asymptomatic catheter-associated thrombosis, as assessed by venography, ranges from 27% to 66% [6]. Common symptoms include pain, swelling, localized warmth, and the development of collateral vessels [7]. As thrombi enlarge, they may detach and embolize to the lungs, resulting in pulmonary embolism. Among long-term cancer patients with central venous access devices, the incidence of pulmonary embolism has been reported to be between 10% and 15% [6], although later studies reported lower rates [8]. Despite the availability of multiple anticoagulant drugs, the rate of recurrent thrombotic events after CRT remains high. In a single-center retrospective study published in 2022, the efficacy of secondary prophylaxis for patients with a prior CVC-venous thromboembolism (VTE) was evaluated in 373 pediatric patients [9]. Exactly 17.4% (65/373) of patients had recurrent VTE, of whom 90.8% (59/65) were CVC-associated. Furthermore, effective anticoagulation against CRT is also complicated by the primary pathologies of patients with catheters, such as patients in intensive care units or those with cancer, renal failure, or pediatric patients [10]. Therefore, it remains imperative to have a better understanding of the pathology of CRT and to develop better and safer therapeutics.

A few recent review papers have comprehensively summarized the incidence, risk factors, diagnosis and clinical management of CRT [8,10,11]. The occurrence of CRT is influenced by both catheter-related and patient-related factors. Catheter-related factors include catheter characteristics, insertion site, and the catheter-to-vein diameter ratio. These factors not only disturb local blood flow and affect endothelial interactions, but also induce direct activation of contact pathway coagulation factors. Patient-related factors, such as malignancy, infection, and a prior history of venous thromboembolism, also affect the risk of CRT. Despite these updates in CRT epidemiology and clinical management, the pathophysiology of CRT remains unclear.

Emerging data indicate that the contact pathway factors XII (FXII) and XI (FXI) might be important mechanistic links between catheter surfaces and blood clotting/CRT. Here, we explicitly review recent evidence on the role of contact pathway factors in the pathobiology of CRT. We summarized up-to-date research on the mechanisms of catheter-promoted activation of FXII and FXI, and importantly, enumerated preclinical and early clinical evidence on the efficacy of contact pathway inhibitors in thrombosis prevention in settings of catheter placement and extra-corporeal circulation systems. Finally, we discuss unresolved questions, including the efficacy and safety of selective FXII/FXI inhibition, as well as novel combination therapies.

We searched relevant literature up to the search date (20 August 2026) in the ClinicalTrials.gov database and PubMed. In particular, the search term (“Contact pathway” OR “Contact activation” OR “FXII” OR “FXI” OR “kallikrein”) AND (Catheter OR Hemodialysis OR ECMO) AND (Thrombosis OR “thrombin generation”) was used in PubMed. In the ClinicalTrials.gov database, we searched interventional trials that match the condition of “catheter OR Hemodialysis OR ECMO” with outcomes of “thrombosis OR clotting”. We included all studies that assessed the antithrombotic effect and safety of contact pathway inhibitors in these settings for analyses, while studies that do not meet these requirements were excluded.

## 2. CRT Risk Factors and Current Therapies

### 2.1. CRT Risk Factors

Catheter characteristics, including type of material, diameter and number of lumens, are key factors influencing the risk of catheter-related thrombosis (CRT) [12]. Synthetic polymers, including polyurethane, silicone, and polytetrafluoroethylene, are currently the primary materials for peripheral venous catheters. Polyethylene and polytetrafluoroethylene catheters have been shown to be more thrombogenic than silicone or polyurethane catheters [12,13,14,15,16,17], while polyurethane and polyvinyl chloride (PVC) exhibit similar thrombogenic potential. Evidence regarding whether silicone is indeed less thrombogenic than polyurethane remains inconsistent [16]. Catheter-to-vein ratio is another important determinant of CRT, and catheters with a larger diameter are also associated with a higher rate of CRT. A retrospective study of hospitalized patients with PICCs found that the risk of CRT with a 5-Fr catheter was 1.8 times higher than that with a 4-Fr catheter, and a 6-Fr catheter increased the risk by 3.9 times [18]. The placement location of catheters also affects the CRT rate. Compared with CVCs, peripherally inserted central catheters (PICCs) are associated with a higher risk of CRT, with an odds ratio (OR) of 2.55 (95% CI 1.54–4.23) [19]. In contrast, implantable ports are associated with the lowest incidence of CRT, reported at only 1–3.8% in cancer patients [19,20], which is significantly lower than the 13.8% incidence observed with PICCs. The higher thrombogenicity of PICC may be attributed to the relatively smaller vein diameter traversed during PICC placement and the longer catheter length. Furthermore, catheter tip position and related endothelial damage also modulate CRT risk [8].

Another major category of CRT risk factors belongs to patient characteristics. Multiple studies have identified a prior history of venous thromboembolism (VTE) as a primary risk factor for CRT [21,22]. A meta-analysis of 5636 cancer patients confirmed that a history of deep vein thrombosis (DVT) significantly increases CRT risk (OR, 2.03; 95% CI 1.05–3.92). Elderly patients also show a notably higher CRT risk compared to younger adults [13]. Malignancy is a widely recognized contributor, promoting CRT through multiple mechanisms, including tissue factor release activating coagulation pathways, tumor compression and immobility causing stasis, and chemotherapy-induced endothelial damage [19,23]. Studies report that cancer patients have a 1.7- to 2-fold higher CRT risk than non-cancer patients [23]. Advanced cancer stage and distant metastasis further elevate CRT risk, with odds ratios of 3.08 and 3.06, respectively [24]. Patients with advanced chronic kidney disease, who often require vascular access for hemodialysis, are also at high risk for CRT due to uremic toxin accumulation and endothelial dysfunction leading to a prothrombotic state. A cross-sectional study of 466 patients receiving their first tunneled hemodialysis catheter reported a CRT incidence of approximately 26.4% [19]. Furthermore, patients with infection have also been shown to have an increased risk of CRT compared with those without [25,26].

### 2.2. Current Interventions Against CRT and Their Drawbacks

**Figure 2:**
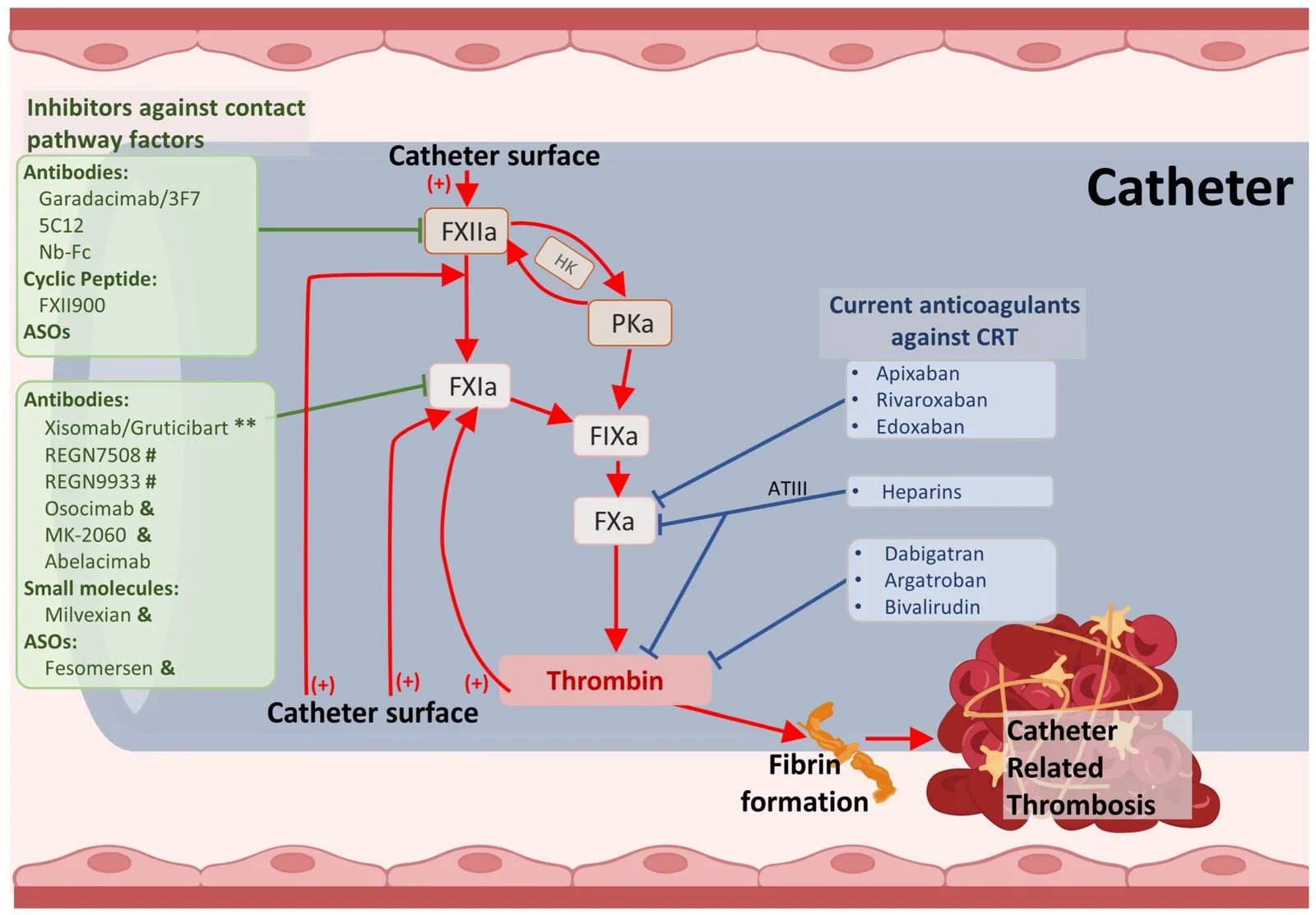
Mechanisms of catheter-associated coagulation activation as well as current and promising therapeutic strategies. Catheters can promote in vitro coagulation activation by inducing a conformational change and the activation of contact pathway factor (F) XII and XI to their activated forms, FXIIa and FXIa, respectively. Catheters have also been shown to augment the activation of FXI by FXIIa and thrombin [34]. The above mechanism accelerates thrombin activation, leads to fibrin clot formation and contributes significantly to catheter-related thrombosis (CRT) pathology. Current anticoagulants against CRT (shown in blue font) rely mainly on heparins, which exert an anticoagulant effect by serving as a cofactor of antithrombin, thereby accelerating its inhibition of thrombin and FXa. Direct oral anticoagulants against FXa and thrombin are also being used for CRT. However, the above medications also impede normal hemostasis potential, thus increase the risk of bleeding. Inhibitors of contact pathway factors XII and XI have been shown to impede catheter-induced thrombin generation and clotting in various in vitro, animal and human trials of catheter placement or extracorporeal circulation systems, without increasing bleeding risk; thus, they may represent promising investigational approaches requiring confirmation in dedicated CRT trials. Note that the inhibitor labeled with ** has shown thrombo-protective efficacy in human trials with CVC; inhibitors labeled with # have been tested in human trials of PICC with results not yet published; inhibitors labeled & with and have been tested in human trials with hemodialysis. The arrows indicate activation. The plus sign indicates augmenting effects. The bar-headed arrows indicate inhibition, Abbreviations: ASOs, antisense oligonucleotides; HK, high molecular weight kininogen; PKa, kallikrein; ATIII, antithrombin.

Anticoagulation therapy remains the primary strategy to address CRT (Figure 2). Current guidelines generally recommend low molecular weight heparin (LMWH) as the first-line treatment, with direct oral anticoagulants (DOACs) and vitamin K antagonists (VKAs) commonly used as alternatives [11]. Routine pharmacological prophylaxis is typically not advised. For patients with symptomatic CRT, anticoagulation is recommended for a minimum duration of three months. Catheter removal should be considered only if dysfunction or severe infection occurs. If the catheter remains in place after three months and the patient has a low bleeding risk, prolonged anticoagulation may be continued until catheter removal.

Low molecular weight heparin (LMWH) is a short-chain derivative of unfractionated heparin. Due to its shorter molecular chain, it mainly targets factor Xa and has less effect on thrombin and other clotting factors. Compared with UFH, LMWH offers significant advantages: predictable pharmacokinetics, generally no need for routine APTT monitoring in most patients [27], once- or twice-daily administration, potent anti-Xa activity, and lower risks of osteoporosis [28]. Multiple guidelines recommend LMWH as the first-line treatment for CRT in cancer patients [11]. Among LMWHs, enoxaparin is widely used clinically, and it has demonstrated superior efficacy in preventing catheter thrombosis compared to apixaban and rivaroxaban [29]. When used at therapeutic doses postoperatively, it lowers CVC-related thrombosis risk with its anti-Xa level positively correlating with antithrombotic efficacy [30].

DOACs consist of direct thrombin inhibitors (e.g., dabigatran) and direct factor Xa inhibitors (e.g., rivaroxaban, apixaban, and betrixaban). These drugs exert their anticoagulant effect by competitively binding to the active site of coagulation factors thrombin or Xa, respectively. DOACs offer several advantages over heparins, including lower risk of drug interactions, relatively short half-lives, fixed dosing regimens, and no requirement for routine coagulation monitoring [31]. In breast cancer patients undergoing chemotherapy via peripherally inserted central catheters, daily administration of 10 mg rivaroxaban combined with handgrip exercises significantly reduced the incidence of catheter-related thrombosis from 12.0% to 2.2%. Statistical analysis confirmed rivaroxaban as an independent protective factor against thrombosis risk [32]. Interestingly, several studies reported an inferior anticoagulant effect of DOACs against catheter-induced thrombin generation compared with heparins in in vitro tests when both were added at therapeutic doses [29]; although, clinical studies observed comparable efficacy between rivaroxaban and standard anticoagulation [2].

Although these anticoagulants can effectively reduce the rate of CRT, they also impede hemostatic potential, thereby increasing bleeding risk [33]. Furthermore, the use of LMWH can cause heparin-induced thrombocytopenia (HIT), although at a lower rate compared with unfractionated heparin [28]. A better understanding of the mechanism of CRT and the development of more effective and safer anticoagulation are highly desired.

## 3. Recent Evidence Supporting the Contribution of Contact Pathway Factors to CRT

### 3.1. The Contact Pathway Factors as Anti-Thrombotic Targets

**Figure 3:**
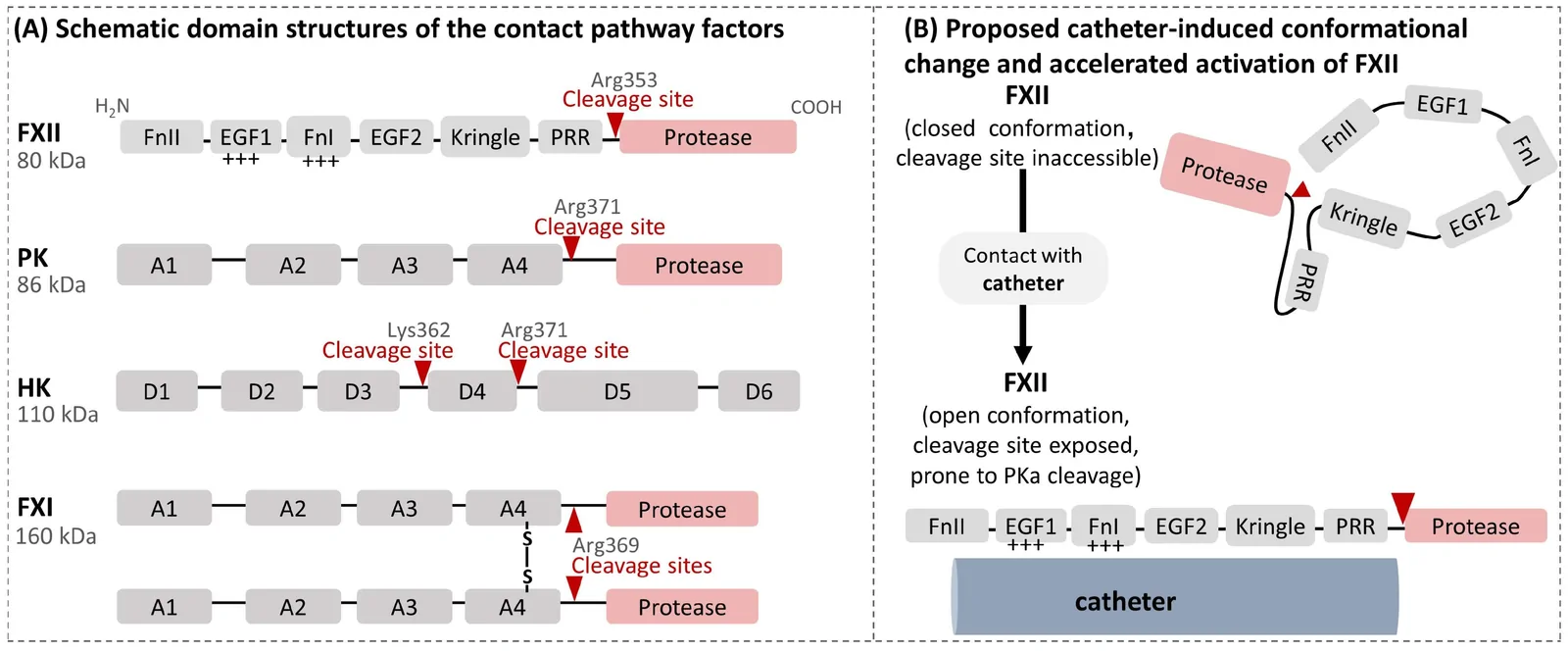
Schematic domain structures of the contact pathway factors and catheter-induced conformational change in FXII. (**A**) Domain structure of FXII, prekallikrein (PK), high molecular weight kininogen (HK) and FXI. (**B**) The proposed mechanism of catheter-induced conformational change in FXII, which makes it more prone to activation by PKa. Recent studies indicate that FXII zymogen is in a closed conformation, in which the activation loop (containing the PKa cleavage site Arg353) is buried by the interactions between the kringle and the FnII domains, and possibly also the protease domain [35]. Upon contact with a catheter, positively charged patches in the EGF1 and FnI domains interact with the catheter surface and result in exposure of the FXII Arg353 cleavage site to enzymes such as PKa. Red arrows indicate the major cleavage sites of the enzymes. The plus signs in (**B**) indicate positively-charged domains.

The contact pathway, or the contact activation system, consists of coagulation factor XII (FXII), prekallikrein (PK), high molecular weight kininogen (HK), and coagulation factor XI (FXI), whose domain structures are depicted in Figure 3.

**FXII** circulates in plasma as a single-chain polypeptide in a closed conformation, which prevents its activation by plasma kallikrein (PKa). Structurally, the mature FXII polypeptide consists of 596 amino acids and comprises seven domains (Figure 3A), including fibronectin type II domain (FnII), epidermal growth factor-like domain 1 (EGF1), fibronectin type I domain (FnI), EGF2 domain, kringle (KNG) domain, proline-rich region (PRR), and a protease domain [36]. Key intramolecular interactions between the FnII, KNG, and catalytic domains collectively shield the Arg353 cleavage site, maintaining the closed state [35,37]. Several positively charged patches in the EGF1 and FnI domains have been shown to be essential for surface-dependent autoactivation of FXII [38,39]. Upon contact with negatively charged surfaces, e.g., polyphosphate (PolyP), cell-free DNA (cfDNA), misfolded protein, or silica, the EGF1 and FnI domains bind to the surface and induce a conformational change in the FXII structure, rendering the cleavage site accessible to PKa [40].

**FXI**, with a plasma concentration of approximately 30 nM, circulates almost entirely bound to HK [41]. Structurally, FXI is a homodimer, each monomer containing four apple domains (A1–A4), and one serine protease domain [42]. The two monomers are linked by a disulfide bond at the Cys321 residue within the A4 domain. The apple domains are responsible for binding ligands such as thrombin, HK, and FXIIa, while the serine protease domain performs the catalytic function.

**HK** is a 626-amino acid polypeptide composed of six domains (D1-D6). Domain 3 is responsible for platelet binding, domain D4 contains the bradykinin (BK) sequence, and the C-terminal domain D6 mediates interactions with PK and FXI [43].

**Plasma prekallikrein (PK)** shares high evolutionary homology with FXI, with 58% amino acid sequence identity [44]. Its structural feature includes four tandem apple domains at the N-terminus, which serve as key binding sites for substrates and cofactors. In the circulation, PK exists as an inactive zymogen tightly bound to HK, forming the PK-HK complex [41]. FXIIa cleaves PK at Arg371, converting it into the enzymatically active PKa. The generated PKa initiates a key positive feedback loop by efficiently activating additional FXII and amplifying the production of FXIIa. This amplification step involves HK and Zn^2+^, thereby strengthening the initial activation signal [45]. Additionally, PK specifically cleaves HK at its D4 domain to produce BK. BK induces significant vasodilation and increased vascular permeability, exacerbating endothelial damage. Around 2020, three groups independently discovered that PKa, activated by FXIIa, dextran sulfate, or an unknown protein on red blood cell-derived extracellular vesicles, can directly activate factor IX (FIX) independent of FXI, leading to thrombin generation in FXI- or FXII- deficient human plasma and mice [46,47,48]. Importantly, although FIX activation by PKa is approximately 2.3-fold more efficient than that by FXIa, considering the much higher plasma concentration of PK than FXI (580 vs. 31 nM), it is reasonable to speculate that PKa may contribute substantially to FIXa activation under certain physiological or pathological settings [49].

Upon exposure to artificial surfaces such as catheters, FXII undergoes conformational changes and is proteolytically converted to α-FXIIa [38], which can be further processed by PKa to generate β-FXIIa [50,51]. α-FXIIa is the primary procoagulant form; it activates FXI and drives thrombin generation and fibrin formation [52,53], thus contributing to CRT. FXIIa promotes the conversion of PK to PKa [54]. The generated PKa further enhances FXII activation, creating a positive feedback loop. PKa also cleaves HK and generates the vasodilator bradykinin.

Contact pathway factors have emerged as promising targets to prevent thrombosis [55]. These factors are found to play important roles in pathological thrombosis triggered by negatively charged surfaces such as catheters (detailed in the next section), while contributing minimally to physiological hemostasis [56,57]. Epidemiological studies indicate that individuals with congenital FXII deficiency do not experience abnormal bleeding. In contrast, a recent study in 703,745 participants discovered that heterozygous carriers of nonsense, frameshift, and essential splice site variants in the *F12* gene (with ~50% reduced circulating FXII level) are protected against venous thromboembolism without an increased risk of bleeding [58]. Congenital FXI deficiency, also known as hemophilia C, is an inherited bleeding disorder that is relatively common in the Ashkenazi Jewish population [59,60]. FXI deficiency is generally well tolerated, with bleeding typically occurring after trauma or surgery; whereas, spontaneous bleeding is rare [61,62]. Conversely, FXI deficiency is associated with a reduced risk of thrombosis, while elevated FXI levels correlate with a higher thrombotic risk [63]. Notably, patients with FXI deficiency have shown acceptable hemostatic safety during procedures involving blood-contacting circuits, such as hemodialysis. Takamizawa et al. reported that a patient with severe FXI deficiency (FXI activity < 3%) and chronic renal failure underwent long-term hemodialysis without heparin anticoagulation and experienced no extracorporeal circuit coagulation or bleeding events [64]. Similarly, another FXI-deficient patient showed no obvious coagulation or bleeding complications during hemodialysis [65]. Overall, it is now generally accepted that inhibition of FXII or FXI could be safe antithrombotic strategies while sparing hemostasis.

### 3.2. Evidence of FXII and FXI Activation by Catheter

Recent studies have proven the pivotal roles of contact pathway factors in CRT. FXII is well-known to undergo autoactivation on certain negatively charged surfaces, including many polymers [66], as well as the titanium surface [67]. In 2011, Yau and colleagues presented convincing evidence for catheter-induced FXII activation and clotting [68]. They found that segments of percutaneous coronary intervention catheters could significantly shorten plasma clotting time, and this procoagulant activity was dependent on FXII. The use of corn trypsin inhibitor (CTI), a specific inhibitor of FXIIa, reduced catheter-induced clotting, while in FXII- or FXI-deficient plasma, the procoagulant effect of catheters was abolished [68]. A later study from the same group showed that reducing FXI or FXII levels in rabbits using antisense oligonucleotides (ASOs) could prolong catheter-induced occlusion time by 2.3-fold and 2.2-fold, respectively, compared with the control group [69].

A recent study revealed that the catheter surface could also promote FXI activation in both direct and indirect manners. Co-incubation of FXI zymogen with the catheter can directly increase its FXIa-like amidolytic activity towards the chromogenic substrate S-2366, suggesting that the catheter can directly activate FXI [34]. Interestingly, the catheter also increased the FXIa-like activity of FXI zymogen that was incubated with its conventional activator, such as thrombin or FXIIa, indicating catheter accelerates FXI activation by these activators. The critical role of FXI in catheter-induced coagulation was further supported by the observation of longer catheter-induced clotting times in FXI-depleted plasma compared with FXII-depleted plasma, in line with an FXII-independent pathway of catheter-induced FXI activation and blood clotting. Currently, the domains responsible for FXI-catheter interaction remain unknown. Catheter placement has been shown to alter the plasma levels of contact pathway-related factors. In vitro studies have shown that exposure of plasma to polytetrafluoroethylene/silicone catheters induces significant elevation of HKa levels, suggesting activation of FXIIa/PKa and consequent HK cleavage [70]. In a rabbit jugular vein catheter model, Malik et al. found that the plasma level of FXIIa–C1 inhibitor complex was significantly increased with catheter placement, indicating marked in vivo activation of FXII [71]. A recent study reported that the first-drawn human blood from newly placed CVCs was hypercoagulable compared with blood obtained after flushing, with significantly higher levels of FVIII and thrombin-antithrombin complex as well as a trend toward lower FXII activity (*p* = 0.16) [72], suggesting rapid activation and clearance of FXII.

## 4. Efficacy of Contact Pathway Inhibition in Thrombosis Prevention in Settings of Catheter Placement and Extracorporeal Circulation Systems

Currently, only a few FXI/FXIa inhibitors have been assessed in the clinical catheter settings, although many other contact pathway inhibitors have been evaluated in in vitro plasma models and animal models of intraluminal catheter occlusion. Relevant clinical and in vivo studies are summarized in Table 1 and Table 2.

Contact pathway inhibitors currently under investigation primarily target FXII and FXI. FXII inhibitors include antisense oligonucleotides (ASOs), monoclonal antibodies, and cyclic peptide inhibitors, which have been mainly evaluated in catheter occlusion models and extracorporeal circulation systems. FXI inhibitors include ASOs, monoclonal antibodies, and small-molecule inhibitors, some of which have advanced to early-phase clinical studies. Small-molecule inhibitors offer the advantage of oral or parenteral administration; whereas, ASOs and antibodies require parenteral delivery. ASOs function by binding to target mRNA and promoting its degradation, thereby indirectly reducing FXI protein levels. However, this approach requires 3–4 weeks of treatment to achieve therapeutic FXI reduction. In contrast, antibodies and small-molecule agents act rapidly, enabling more immediate anticoagulant control.

### 4.1. Clinical Trials of FXI/FXIa Inhibitors for CRT

Although both viewed as safe anticoagulant targets, several studies show that FXI inhibition tends to be more effective than FXII inhibition at reducing artificial surface-induced thrombosis [85,86]. This may be explained by the dual role of FXI in the coagulation cascade: it not only mediates FXIIa-initiated thrombin generation but also amplifies thrombin production via the thrombin–FXI feedback loop [87,88]. As recently reviewed by Capodanno et al., when evaluated in settings of total knee arthroplasty (TKA), FXI/FXIa inhibitors Milvexian, Abelacimab and Osocimab achieved VTE protection similar to or better than that of enoxaparin, with no increase in bleeding (AXIOMATIC-TKR, ANT-005, FOXTROT) [89]. Human data directly evaluating contact pathway inhibition for CRT prevention remain extremely limited. To date, only a small number of early-phase clinical studies have investigated FXI-directed strategies in patients with central venous catheters.

**Gruticibart** (a.k.a. **AB023** or **Xisomab**), a humanized version of the 14E11 antibody, binds specifically to FXI and inhibits its activation by FXIIa while preserving thrombin-mediated FXI activation [90]. A phase II prospective study [73] evaluated the efficacy of a single dose of 2 mg/kg Xisomab within 24 h of CVC placement in 22 ambulatory cancer patients (NCT04465760) and found no drug-related serious adverse events among the nine treated patients. Surveillance ultrasound also revealed a reduction in rates of asymptomatic, nonocclusive mural thrombosis from 40% in the control group to 12.5% in the intervention group on day 14 post CVC placement. Unlike patients on placebo, patients on Gruticibart did not have increased level of thrombin-antithrombin complex (a biomarker for in vivo coagulation activation). Gruticibart also reduced plasma levels of FXIa-AT complex. Another phase II study (NCT07498517) was initiated in early 2026 to investigate the effect of Gruticibart in reducing CRT among patients with a CVC) inserted.

**REGN9933** and **REGN7508** are monoclonal antibodies targeting the apple 2 domain and the catalytic domain of FXIa, respectively. A phase 2 randomized, double-blind, placebo-controlled study, named ROXI-CATH (NCT06299111), evaluating the efficacy of these antibodies for thrombosis prevention in patients with PICCs completed participant enrollment in early 2026; results have yet to be posted. A phase III trial of these agents in the same setting of PICC has also been initiated (NCT07697599), with participant enrollment yet to be started.

### 4.2. Human Trials of FXI Inhibition in Extracorporeal Circulation Systems

The use of extracorporeal circulation systems (such as ECMO and hemodialysis circuits) is also associated with significant activation of coagulation and thrombosis [1]. These settings also involve contact pathway activation induced by artificial surfaces. Representative in vivo findings from related studies are also included here. However, it is important to note that the sites of thrombosis and clinical phenotypes differ across models; ECMO and hemodialysis devices involve larger artificial surface areas and different hemodynamic conditions. Catheter occlusion models primarily assess catheter dysfunction caused by intraluminal thrombosis; whereas, CRT focuses on venous thrombosis at the catheter site. Therefore, while these findings support the notion that contact pathway inhibition confers thrombosis protection from artificial surfaces, they cannot be directly extrapolated to clinical CRT prevention and should be interpreted with caution.

Several human trials have tested FXI/FXIa inhibitors in patients undergoing hemodialysis. In a completed phase II trial [74] involving 24 patients with end-stage renal disease (ESRD) undergoing heparin-free hemodialysis (NCT03612856), a single pre-dialysis dose of 0.5 mg/kg FXI antibody **AB023** was associated with an ~60% reduction in the requirement for hemodialysis circuit replacement or flush, a result not seen in the placebo group. AB023 use was not associated with impaired hemostasis or other drug-related adverse events.

**Fesomersen** (**IONIS-FXI-LRx)**, an ASO targeting FXI mRNA, has been evaluated in patients with end-stage renal disease (ESRD) requiring hemodialysis. A phase II trial [75] demonstrated that Fesomersen effectively reduced FXI activity. By day 85, mean levels of FXI activity fell 56.0% in the 200 mg group, 70.7% in the 300 mg group. IONIS-FXI-LRx was not associated with drug-related serious adverse events. One major bleeding event was reported but was considered unrelated to treatment. In a separate phase II study [76] involving 307 patients with kidney failure on hemodialysis (NCT02553889), Fesomersen similarly produced dose-dependent reductions in FXI levels by 54% to 86%. Lower predicted FXI levels were significantly associated with reduced hemodialysis circuit clotting (*p* = 0.002) and AV-access thrombosis (*p* = 0.014), while the incidence of major or clinically relevant non-major bleeding was comparable between the Fesomersen and placebo groups.

In a phase II double-blind, placebo-controlled CONVERT trial [77] involving 704 patients with kidney failure undergoing hemodialysis (NCT04523220), **Osocimab** (**BAY-1213790**), a humanized monoclonal antibody against FXIa, was given 105 or 210 mg monthly followed by half dose for maintenance. The use of Osocimab exhibited a bleeding risk comparable to placebo (6.9 and 4.9% vs. 7.8%). Meanwhile, Osocimab was associated with a reduction in the exploratory efficacy outcome of the incidence of arteriovenous fistula or graft thrombosis over the main treatment period: the cumulative incidence risks were 1.7% (90% CI, 0.70–3.61) for lower-dose Osocimab, 2.68% (90% CI, 1.29–4.91) for higher-dose Osocimab and 3.91% (90% CI, 2.18–6.43) for placebo.

**MK-2060**, another monoclonal antibody targeting FXI, has also been evaluated in a randomized parallel-group, placebo-controlled, double-blind, multi-center phase II trial for the prevention of thrombosis in 506 patients with end-stage renal disease receiving hemodialysis (NCT05027074). Patient recruitment was completed in early 2026. MK-2060 at doses of 6 or 20 mg did not mitigate the primary outcome measure: the time to first arteriovenous graft (AVG) thrombosis event, with HR of 0.87 (95% CI: 0.60 to 1.26) and 0.83 (95% CI: 0.57 to 1.19) when comparing the high or low MK-2060 dose groups with the placebo group, respectively.

### 4.3. Other FXI/FXIa Inhibitors Under Early Evaluation for MDAT

**Milvexian** (**BMS-986177** or **JNJ-70033093**), a small-molecule inhibitor of FXIa, demonstrated an overall favorable safety and tolerability profile in a phase I study [91]. Milvexian added at 600 ng/mL inhibited in vitro thrombin generation in catheter-incubated human platelet-rich plasma, reducing peak thrombin level by ~60% [92]. Yin et al. reported that the combination of heparin and Milvexian synergistically inhibited catheter-induced thrombin generation, with effects exceeding the simple additive outcomes of each drug alone [34]. A randomized phase I/II trial in 2017 (NCT03000673) evaluated the safety of Milvexian in 32 ESRD patients on hemodialysis. Milvexian at doses of 100 or 300 mg was compared with 40 mg enoxaparin or UFH; no mortality or serious adverse events were found in any group. Its antithrombotic efficacy remains to be evaluated.

**Abelacimab** (**MAA868**) is a humanized IgG1 monoclonal antibody that inhibits both FXI and FXIa, blocking activation by FXIIa and thrombin [93]. Abelacimab added at 50 μg/mL inhibited in vitro thrombin generation in catheter-incubated human platelet-rich plasma, reducing peak thrombin level by ~80% [92]. However, its efficacy in preventing CRT has not been tested in human trials.

### 4.4. FXII Inhibition in Animal Models of Intraluminal Occlusion and Catheter-Induced In Vitro Coagulation

FXII is activated to FXIIa when activated by negatively charged materials; FXIIa then converts PK to PKa, which cleaves HK to release the vasodilator bradykinin. Due to its key role in the activation of the kallikrein–kinin system, FXII is thus a useful target to treat hereditary angioedema (HAE), a critical condition characterized by aberrant generation of bradykinin and systemic swelling of the skin/subcutaneous tissues, gastrointestinal tract, and upper airway. The FXII antibody 3F7 blocks kallikrein–kinin system activation [94,95]; its modified version, **Garadacimab (CSL312)**, has successfully completed a phase III clinical trial (NCT03712228) [96] and has been approved for HAE. A clinical trial in patients with severe COVID-19 evaluating the effect of Garadacimab plus standard of care on endotracheal intubation or death confirmed its favorable safety profile, with no increased bleeding risk observed [97]. However, FXII inhibition has not been evaluated for thrombosis protection in human MDAT trials, although it has been extensively assessed in in vitro and animal models.

FXII-targeted approaches have been evaluated in several animal ECMO models. FXII knockdown with ASOs prolonged ECMO circuit lifespan [78], although its efficacy remains to be validated in primate models or human studies. In a rabbit ECMO model, 3F7, a humanized monoclonal antibody that binds the protease domain of FXII and inhibits FXIIa enzymatic activity, suppressed pathological thrombosis as effectively as heparin, without increasing bleeding from wounds [80], supporting the antithrombotic potential of FXIIa inhibition in extracorporeal blood-contacting systems. Several other FXIIa inhibitors, including the mAb **5C12** and the cyclic peptide inhibitor **FXII900**, have also been evaluated in animal ECMO models [81,83]. Both agents consistently exhibited significant antithrombotic activity with a favorable safety profile.

FXII inhibition has also shown favorable outcomes in animal catheter occlusion models. Genetic knockdown of FXII levels with **FXII-ASOs** significantly prolonged catheter patency in rabbit intraluminal catheter occlusion models [69,71]. The anti-FXII antibody Garadacimab has also been evaluated in in vitro models of catheter-induced thrombin generation in human platelet-rich plasma. **Garadacimab**, at a clinically relevant dose of 50 μg/mL, prolonged the lag time of thrombin generation from ~8 min to ~25 min and reduced peak thrombin level by ~66% [92], supporting the ability of FXIIa inhibition to attenuate catheter-induced coagulation in vitro. Additionally, preventing surface-induced FXII autoactivation could represent a novel strategy to reduce catheter-associated coagulation. Frunt et al., recently identified two distinct positively charged amino acid patches within the EGF1 domain of FXII as essential for FXII binding to negatively charged materials [98]. A nanobody **F2**, by neutralizing the charge of these patches, was shown to almost completely abolish FXII binding to kaolin surfaces and reduce autoactivation by 94%. The thrombo-protection efficacy of this approach remains to be explored in CRT models.

Furthermore, multitargeted contact pathway inhibitors targeting both FXII and FXI have also been investigated in intraluminal catheter occlusion models. **Ixodes ricinus-contact phase inhibitor** (**Ir-CPI**), a recombinant protein expressed in tick salivary glands, has demonstrated potent anticoagulant efficacy in a rabbit catheter occlusion model, with an antithrombotic effect comparable to that of heparin, yet not associated with increased bleeding [84].

## 5. Unresolved Questions and Future Directions

### 5.1. Can FXII/FXI Inhibitors Completely Suppress Catheter-Induced Clotting and CRT?

Although experimental data indicate catheter surfaces can induce blood coagulation through activation of FXII and FXI [34,69], recent studies reveal that even high concentrations of FXI inhibitors do not completely suppress catheter-induced coagulation activation. A recent study showed that the FXIIa inhibitor Garadacimab, or the FXI inhibitors such as Abelacimab, Asundexian, and Milvexian, when used alone at high doses, failed to completely suppress catheter-induced thrombin generation in platelet-rich plasma [92]. In contrast, high doses of enoxaparin, UFH, or dabigatran can achieve full suppression. Importantly, combined use of low-dose Milvexian with unfractionated heparin also results in a complete inhibition of catheter-induced plasma thrombin generation [34]. These observations point to a possibility that catheter-induced coagulation may be mediated through direct activation of coagulation factors other than FXII or FXI.

A 2024 study identified that plasma kallikrein (PKa), activated by silica, can induce thrombin generation independent of FXII [49]. In whole blood from FXII-deficient mice, PKa inhibition with the small-molecule inhibitor Berotralstat or an antibody Lanadelumab significantly reduced silica-initiated thrombin generation [49]. Given that catheters can directly activate FXI [34], and that PK shares high structural and sequence similarity with FXI [44], it is possible that catheter could directly activate PK, and the resulting kallikrein (PKa) might then directly activate factor IX [47] thereby bypassing FXII and FXI. Currently, several drugs targeting PK/PKa have been approved for the prevention of hereditary angioedema (HAE), including Donidalorsen (an ASO that reduces prekallikrein expression), Lanadelumab (a monoclonal antibody targeting activated plasma kallikrein), and Berotralstat (an oral small-molecule plasma kallikrein inhibitor). However, the experimental anticoagulant effects of PKa inhibitors have not yet translated into clear antithrombotic signals in clinical settings. Multiple studies in HAE patients have confirmed that contact pathway overactivation (PKa, FXIIa, FXIa) due to C1-inhibitor deficiency is associated with an increased thrombotic risk [99,100], and that C1-inhibitor replacement therapy, by simultaneously inhibiting multiple contact system proteases, can effectively reduce this risk [101]. In contrast, although extensive clinical experience shows that PK/PKa-targeting drugs effectively suppressed PKa activity, no clear antithrombotic benefit has been observed to date, nor an increase in bleeding risk. The lack of a clear antithrombotic effect with PKa inhibition could be due to that the FXIIa–FXI axis remains capable of sustaining coagulation despite PKa inhibition. Therefore, whether PKa inhibition can exert a clinically meaningful antithrombotic effect at the catheter–blood interface remains uncertain and warrants further validation.

### 5.2. Limitations of Current Evidence for the Role of Contact Pathway Factors in CRT

Overall, several lines of evidence have been reported to support a potential role of contact pathway factors XII and XI in CRT, including (1) mechanistic in vitro evidence, (2) animal models of catheter-occlusion and extracorporeal circulation and (3) human catheter studies; however, several limitations should be noted. First, the evidence summarized here are from studies that uses heterogenous models that, despite share similarity of surface-induced contact activation, differs in hemodynamic conditions and sites of thrombus formation, and therefore should be interpreted with caution. Human trials on CRT remain scarce. Currently, only one trail reported the protective effect of FXI inhibition against a surrogate endpoint (ultrasound-detected asymptomatic mural thrombosis) at day 14 post CVC placement [73]. Larger phase III trials are needed to verify whether this approach provides protection against symptomatic thrombosis.

Second, the relative contribution of FXII/FXI-mediated contact activation in CRT remains unclear when compared with other established risk factors, including patient-specific thrombotic predisposition, catheter-related mechanical factors and endothelial injury [8]. Importantly, FXI inhibition with Gruticibart did significantly reduce the rates of asymptomatic mural thrombosis in 22 cancer patients [73], suggesting FXI plays an important role in CRT, even in the complex setting of malignancy that are known to have various pro-thrombotic risk factors [23]. Interestingly, in vivo FXI activation has been associated with cancer-associated thrombosis. In a 719-patient prospective NSCLC cohort (HYPERCAN), baseline activated FXI–antithrombin (FXIa–AT) complexes independently predicted 6-month VTE (sHR 1.17, 95% CI 1.00–1.37) and mortality [102]. However, future studies are needed to examine whether FXII/FXI inhibition also exerts protection against CRT in patients with other thrombosis-predisposing risk factors. Aside from contact pathway inhibition, efforts should also be spent on exploring strategies to mitigate catheter-related risk factors, including catheter-to-vein ratio optimization, catheter material engineering or antithrombotic surface coatings. Additionally, patient-specific risk stratification strategies should be explored to aid more effective and safer pharmaceutical interventions.

Furthermore, although in vitro studies showed that catheter can directly promote activation of FXII and FXI, it remains unclear whether contact activation is an initiating event or a secondary amplifier in human CRT. FXI inhibition with Gruticibart did significantly reduce the rates of asymptomatic mural thrombosis in 22 cancer patients [73], suggesting that FXI plays an important role in CRT. However, this observation cannot answer the above question, since FXI involves in both the classical contact activation pathway (FXIIa-FXI) and the feed-back loop (Thrombin-FXI). Trials testing the effect of FXII inhibition in human CRT would help answer this question.

### 5.3. Really No Side-Effects When Inhibiting Contact Pathway Factors?

Although generally viewed as unnecessary for hemostasis, FXII and FXI have been recently shown to play vital roles in protection against infection. Nickel et al. demonstrated that FXII-driven coagulation enhances innate immunity by trapping pathogens and restricting bacterial infection in mice [103]. In mouse models of Streptococcus pneumoniae intranasal inoculation or Staphylococcus aureus skin infection, FXII-deficient mice had more severe infection, with increased bacterial burden, systemic spread, and mortality. FXI-deficient mice had similarly worsened outcomes against infection. In contrast, a recent analysis of 703,745 participants by Haj et al. found that individuals with FXII haploinsufficiency (~50% reduced plasma FXII level) did not have an increased risk of infection [58]. However, it remains unknown whether a higher degree of inhibition of FXII (e.g., >90%) would affect protection against infection. Therefore, infection still needs to be closely monitored when FXII inhibitors are used, especially considering that a large number of patients with catheters are in severe conditions with compromised immune systems.

Although FXI inhibitors have been found to have favorable safety profiles compared to DOACs when used for stroke prophylaxis in patients with atrial fibrillation [104], potential effects of FXI inhibition on cardiac function warrant attention. Cao et al., [105] reported that the proteolytic activity of FXI is required for the cleavage and activation of extracellular matrix-associated BMP7 in the heart, thereby inhibiting genes involved in inflammation and fibrosis. In mouse models of diet-induced heart failure with preserved ejection fraction (HFpEF), FXI level was inversely correlated with the extent of diastolic dysfunction, with greater expression of FXI reducing cardiac fibrosis and inflammation. Corresponding clinical observations showed that plasma FXI levels were higher in patients with HFpEF, suggesting a protective role of FXI in diastolic function. Another analysis of cohorts from ARIC (Atherosclerosis Risk in Communities) and CHS (Cardiovascular Health Study) also observed significant associations between plasma FXI level and cardiac function [106]. Lower FXI level was associated prospectively with higher incidence of heart failure, as well as higher prevalence of diastolic dysfunction and worse E/A ratio, left atrial function, and left ventricular mass index. However, a recent report by Daghlas et al., who analyzed the deCODE cohort (N = 35,559), found that individuals with an F11 variant that reduced circulating FXI levels by 0.33 SD units did not have elevated all-cause heart failure (odds ratio, 0.99 [95% CI, 0.96–1.01]; *p* = 0.34), or with any secondary clinical or radiographic outcomes [107]. Nevertheless, these conflicting observations call for close monitoring of the long-term safety of FXI inhibitors in larger populations and over extended follow-up periods.

In conclusion, several lines of evidence support the important role of contact pathway factors in the pathology of CRT. In vitro studies revealed that catheters can promote the activation of FXII and FXI. Preclinical and early clinical studies demonstrate that agents targeting FXII or FXI could reduce catheter-induced in vitro plasma clotting, as well as intraluminal clotting in animal models of catheterization or ECMO. Although clinical data remain quite limited currently, FXII/FXI inhibition are well-tolerated and generally exert favorable antithrombotic effects in human trials of CVC placement or hemodialysis. Considering the complex pathology of CRT involving both catheter-related and patient-related risk factors, larger phase III human trials are needed to further confirm the antithrombotic efficacy of FXII/FXI inhibition in CRT and the relative importance of the contact pathway in CRT. Although clinical data from the HAE trials [96] and TKA trials [89] demonstrate that inhibition of FXII or FXI, respectively, does not increase bleeding risk, attention should be paid for FXII inhibitors on long-term infection risk [103] in immunocompromised patients with catheters. Despite that conflicting findings have been reported [105,106,107], potential side-effects on cardiac remodeling and diastolic function associated with FXI inhibition should be further verified. Lastly, considering that a combination of low doses of FXI inhibitors and heparin can more effectively abolish catheter-induced in vitro plasma clotting compared with the single use of either agent [34], it would be worthwhile to test this strategy in human trials to explore whether it could deliver better efficacy, safety and cost-efficacy profiles in CRT management.

## Figures and Tables

**Table 1:** Clinical studies of contact pathway inhibitors in the settings of catheter placement and hemodialysis.

Agent; Type	Target	Mechanism and Estimated Half-Life	Clinical Settings and Interventions	Clinical Trial Identifier and Results	Ref.
Gruticibart (Xisomab /AB023); Antibody	FXI	targets the apple 2 and 4 domains of FXI, blocking FXIIa-mediated but not thrombin-mediated FXI activation; t_1/2_ 11–121 h	CVC use in ambulatory cancer patients n = 22; 2 mg/kg Gruticibart or placebo	Phase II; (NCT04465760); Completed; Gruticibart group with lower rates of asymptomatic, nonocclusive mural thrombosis 12.5% vs 40%	Pfeffer, 2024 [73]
			CVC use; Gruticibart or placebo	Phase II; (NCT07498517); Not yet recruiting	NCT07498517
			ESRD on hemodialysis, n = 24; 0.25 or 0.5 mg/kg AB023 or placebo	Phase II; (NCT03612856); Completed; Gruticibart reduced the needs for hemodialysis circuit replacement or flushing by ~60%, vs 0% in placebo group	Lorentz, 2021 [74]
REGN7508 and REGN9933; Antibody	FXI/FXIa	targeting the apple 2 and the catalytic domains of FXI(a), respectively	Adult and adolescent with PICC; Antibody or placebo	Phase II; (NCT06299111); Completed but data not disclosed yet	NCT06299111
			Adult and adolescent with PICC; Antibody or placebo	Phase III; (NCT07697599); Not yet recruiting	NCT07697599
Fesomersen (IONIS-FXI-LRx; BAY2976217); ASOs (s.c.)	FXI	binds to FXI mRNA and promotes its degradation, thereby reducing FXI protein levels; t_1/2_ 10–20 d	patients with ESRD on hemodialysis, n = 49; 200 mg or 300 mg IONIS-FXI-LRx or placebo	Phase II; (NCT04534114); Completed; 1 treatment-irrelevant major bleeding in both the ASO and placebo groups	Walsh, 2022 [75]
			kidney failure on hemodialysis, n = 307; Fesomersen 40, 80, or 120 mg once monthly, or placebo	Phase II; (NCT02553889); Completed; Fesomersen did not increase major bleeding or major atherothrombotic events.	Winkelmayer, 2024 [76]
Osocimab (BAY-1213790); Antibody	FXIa	specifically binds to FXIa and inhibits its coagulant activity; t_1/2_ 30–44 d	Patients with kidney failure on hemodialysis, n = 704; 105 or 210 mg Osocimab monthly followed by half dose for maintenance, compared with placebo	Phase II; (NCT04523220); Completed; Osocimab was not associated with increased bleeding, but with a reduction in the risk of moderate-to-complete dialysis circuit clotting compared with placebo	Weitz, 2024 [77]
MK-2060; Antibody	FXI	Anti-FXI	ESRD receiving hemodialysis, n = 506; 6 or 20 mg MK-2060 or placebo	Phase II; (NCT05027074); Completed; MK-2060 did not mitigate the time to first arteriovenous graft (AVG) thrombosis event.	NCT05027074
Milvexian (BMS-986177/ JNJ-70033093); Small molecule	FXIa	directly inhibits the enzymatic activity of FXIa; t_1/2_ 11–18 h	ESRD on hemodialysis, n = 32; 100 or 300 mg Milvexian or 40 mg Enoxaparin or UFH	Phase I, II; (NCT03000673); Completed, no data on thrombo-protection efficacy	NCT03000673

Abbreviations: FXI, factor XI; CVC, central venous catheter; PICC, peripherally inserted central venous catheters; ESRD, end-stage renal disease; UFH, unfractionated heparin.

**Table 2:** Preclinical studies of contact pathway inhibitors in animal models of intraluminal catheter occlusion and extracorporeal systems.

Type	Anticoagulants	Settings	Outcomes	Ref
**Target: FXI**				
ASO	ISIS 564673	Rabbit intraluminal catheter occlusion model	prolongs catheter occlusion time 2.3-fold	Yau, 2014 [69]
	FXI ASO	VA-ECMO in rabbits	prolongs ECMO circuit lifespan and prevents fibrinogen consumption	Tweddell, 2023 [78]
Antibody	Osocimab	ECMO in baboons	reduces platelet and fibrin deposition in oxygenators; outperforms LMWH in ex vivo models by better preserving platelet function	Samaha, 2025 [79]
**Target: FXII**				
ASO	ISIS 564859	Rabbit intraluminal catheter occlusion model	prolongs catheter occlusion time by 2.2-fold	Yau, 2014 [69]
	FXII ASO	Rabbit intraluminal catheter occlusion model	Significantly prolongs catheter occlusion time	Malik, 2023 [71]
	FXII ASO	VA-ECMO in rabbits	extends ECMO circuit lifespan, reduces pulmonary edema and bleeding, compared with heparin	Tweddell, 2023 [78]
Antibody	3F7	ECMO in rabbits	prevents thrombosis as effectively as heparin but with less wound bleeding	Larsson, 2014 [80]
	5C12	ECMO in rabbits	reduces platelet and fibrin deposition in oxygenators without increasing bleeding	Wallisch, 2020 [81]
	Nb–Fc	ECMO in mice	reduces oxygenator thrombosis and systemic microvascular thrombosis while attenuating thromboinflammation	Xu, 2024 [82]
Cyclic peptide	FXII900	ECMO in rabbits	suppresses coagulation in extracorporeal circuits without increasing bleeding risk	Wilbs, 2020 [83]
**Target: FXII and FXI**				
Protein Inhibitor	Ir-CPI	Rabbit intraluminal catheter occlusion model	prevents circuit coagulation as effectively as heparin, without promoting bleeding	Pireaux, 2019 [84]

Abbreviations: FXI, factor XI; ASO, antisense oligonucleotide; VA, venoarterial; ECMO, extracorporeal membrane oxygenation; FXII, factor XII.

## Data Availability

No new data were created or analyzed in this study. Data sharing is not applicable to this article.
